# The York County Hospital

**Published:** 1913-03-15

**Authors:** 


### THE YORK COUNTY HOSPITAL.

### Letter from Dr. W. MOIR SHEPHERD, late House Physician, and Dr. J. GORDON MACQUEEN, late House Surgeon.

We have received the following statement signed by J. Gordon Macqueen, M.B., Ch.B. Edin., and W. Moir Shepherd, M.B., Ch.B. Edin., who, until the end of January 1913, occupied the position of resident medical officers at the York County Hospital, York. It may be in the remembrance of our readers that in The Hospital of January 27, 1912, pp. 433-4, we published a report of a careful inspection of this institution and that that report plainly intimated to the Committee and Governors that the state of the management was such that it demanded their immediate and careful attention. How precisely accurate and justified this diagnosis of our Commissioner was will be plain from the knowledge that the events described by Drs. Shepherd and Macqueen have taken place subsequently to the publication of our Commissioner's report.

Our sole object in incurring the grave responsibility of giving publicity to their report is that they came to us on the recommendation of their Professor in the great school of medicine where they were trained, that the Committee of the York County Hospital have taken no effective notice whatever of the friendly warning in our Commissioner's report, and that they have permitted the general administration of the internal affairs of the hospital to continue to be conducted in circumstances which rendered possible the existence of the grave state of matters which the following report exhibits.

Our columns are open to the authorities of the York County Hospital, and we shall be glad to give them ample space if they will avail themselves of it, in order that they may be in a position to secure equal publicity for their case and reply.

